# A robust deep learning framework for cerebral microbleeds recognition in GRE and SWI MRI

**DOI:** 10.1016/j.nicl.2025.103873

**Published:** 2025-08-27

**Authors:** Tahereh Hassanzadeh, Sonal Sachdev, Wei Wen, Perminder S. Sachdev, Arcot Sowmya

**Affiliations:** a School of Computer Science and Engineering, University of New South Wales, Sydney, NSW 2052, Australia; b Prince of Wales Hospital, Neuropsychiatric Institute, Sydney, NSW, Australia; c Centre for Healthy Brain Ageing (CHeBA), Discipline of Psychiatry and Mental Health, School of Clinical Medicine, UNSW Medicine, Sydney NSW 2052, Australia; d St Vincent’s Hospital, Medical Imaging Department, Sydney, Australia

**Keywords:** Cerebral microbleeds detection, Deep learning, GRE MRI, SWI MRI, 3D CNN, YOLO

## Abstract

Cerebral microbleeds (CMB) are small hypointense lesions visible on gradient echo (GRE) or susceptibility-weighted (SWI) MRI, serving as critical biomarkers for various cerebrovascular and neurological conditions. Accurate quantification of CMB is essential, as their number correlates with the severity of conditions such as small vessel disease, stroke risk and cognitive decline. Current detection methods depend on manual inspection, which is time-consuming and prone to variability. Automated detection using deep learning presents a transformative solution but faces challenges due to the heterogeneous appearance of CMB, high false-positive rates, and similarity to other artefacts. This study investigates the application of deep learning techniques to public (ADNI and AIBL) and private datasets (OATS and MAS), leveraging GRE and SWI MRI modalities to enhance CMB detection accuracy, reduce false positives, and ensure robustness in both clinical and normal cases (i.e., scans without cerebral microbleeds). *A 3D convolutional neural network (CNN) was developed for automated detection, complemented by a You Only Look Once (YOLO)-based approach to address false positive cases in more complex scenarios.* The pipeline incorporates extensive preprocessing and validation, demonstrating robust performance across a diverse range of datasets. The proposed method achieves remarkable performance across four datasets, ADNI: Balanced accuracy: 0.953, AUC: 0.955, Precision: 0.954, Sensitivity: 0.920, F1-score: 0.930, AIBL: Balanced accuracy: 0.968, AUC: 0.956, Precision: 0.956, Sensitivity: 0.938, F1-score: 0.946, MAS: Balanced accuracy: 0.889, AUC: 0.889, Precision: 0.948, Sensitivity: 0.779, F1-score: 0.851, and OATS dataset: Balanced accuracy: 0.93, AUC: 0.930, Precision: 0.949, Sensitivity: 0.862, F1-score: 0.900. These results highlight the potential of deep learning models to improve early diagnosis and support treatment planning for conditions associated with CMB.

## Highlights

• We propose a patch-based 3D CNN for detecting cerebral microbleeds (CMB).
• Various scenarios are considered to address the challenges of both large and small datasets.
We provide guidelines for configuring the model to prioritise higher accuracy or robustness in detecting CMB, particularly in normal cases.
• To our knowledge, this is the first study leveraging the ADNI dataset for automatic CMB detection.
• The model achieves a remarkably low false positive rate, lower than one per scan in the test set for both GRE and SWI datasets.

## 1 Introduction

Cerebral microbleeds (CMB) are small, dark spots or lesions seen on gradient echo (GRE) or susceptibility-weighted (SWI) MRI scans, indicating localised deposits of hemosiderin due to tiny brain haemorrhages. *GRE (Gradient Recalled Echo) is a conventional T2*-weighted MRI sequence that is widely used in clinical settings. It is sensitive to magnetic susceptibility effects, making it helpful in detecting CMBs, although it may offer lower spatial resolution and contrast compared to more advanced techniques. SWI (Susceptibility-Weighted Imaging), on the other hand, is a high-resolution T2*-based sequence that combines both magnitude and phase information to enhance the visibility of small haemorrhages and venous structures. Due to its higher sensitivity and contrast, SWI is considered more effective than GRE in detecting small or subtle CMBs. The CMBs serve as critical indicators of several cerebrovascular conditions such as vascular dementia, small vessel disease, cerebral amyloid angiopathy and Alzheimer’s disease (Al-Masni et al., 2020). These lesions are linked to higher risks of cognitive decline, neurological impairment and long-term disability, underscoring the importance of accurate detection and characterisation for effective patient management and treatment planning. Accurate detection and quantification of cerebral microbleeds (CMB) are crucial for guiding anticoagulation therapy, anti-amyloid antibody treatment, surgical planning and managing cognitive decline. This is especially important as a higher count of microbleeds is associated with increased vascular pathology and hemorrhagic risks. For example, a recent study (Cummings et al., 2023) categorised patients into three groups to determine the appropriate dosage of Lecanemab in the ARIA (amyloid-related imaging abnormalities) (Roytman et al., 2023) study, which aims to treat individuals in the early stages of Alzheimer’s disease. In other work (Cummings et al., 2023), patients with fewer than four CMB, five to nine CMB, and 10 or more CMB were classified as mild, moderate and severe cases, respectively. Treatment was discontinued if the number of CMB in severe cases increased to more than 10 after starting treatment (Cummings et al., 2023). This highlights the critical need for precise detection and accurate quantification of CMB to ensure effective treatment decisions and avoid complications. In addition, ensuring that there are no false positives in normal individuals is essential to avoid unnecessary interventions and anxiety. Moreover, CMB detection supports research into disease mechanisms, progression and treatment efficacy, improving patient care while minimising healthcare burdens. Detecting CMB is a significant challenge due to its small size, rarity, and the possibility of numerous CMB mimics across hundreds of MRI slices. These mimics, including calcifications, flow voids, or imaging artefacts, significantly complicate detection efforts.

Currently, the standard method for identifying CMB relies on visual examination by trained radiologists, a process known for its subjectivity, time intensity and variable reliability (Chesebro et al., 2021). Automated detection techniques using deep learning have emerged as a promising strategy to mitigate these challenges, aimed at enhancing the Precision and efficiency of diagnosis (Ferlin et al., 2023).

This study examines deep learning techniques for CMB detection, aiming to enhance detection performance and minimise false positives, particularly in typical cases. By “normal cases,” we mean subjects whose MRI scans do not show any cerebral microbleeds (CMB). After comprehensive data preparation, a 3D patch-based CNN is developed for CMB detection. To address data imbalance due to the rarity of CMB, various approaches have been proposed, including using all data, undersampling, and employing the YOLO (You Only Look Once) model (Redmon, 2016), to increase CMB detection accuracy and reduce false positives. We selected multiple datasets, including the Alzheimer’s Disease Neuroimaging Initiative (ADNI) GRE dataset that contains hundreds of MRIs and thousands of CMB, and a subset of the Australian Imaging Biomarkers and Lifestyle Study of Ageing (Ellis et al., 2009) (AIBL) SWI dataset. In addition, two SWI datasets from the Centre for Healthy Brain Ageing (CHeBA), namely the Sydney Memory and Ageing Study (MAS) and Older Australian Twins Study (OATS), were also utilised. The results consistently demonstrated high accuracy, robustness across imaging modalities, and effectiveness on small and large datasets. The proposed framework proved adaptable, delivering consistently strong performance with reduced false positives, regardless of dataset size. By automating CMB detection, this research contributes to the broader goal of personalised medicine and Precision healthcare in neurology.

The proposed contributions may be summarised as follows:

1. We propose a patch-based 3D CNN for detecting cerebral microbleeds (CMB).
2. Various scenarios are considered to address the challenges of both large and small datasets.
3. We provide guidelines for configuring the model to prioritise higher accuracy or robustness in detecting CMB, particularly in normal cases.
4. To our knowledge, this is the first study leveraging the ADNI dataset for automatic CMB detection.
5. The model achieves a remarkably low false positive rate, lower than one per scan in the test set for both GRE and SWI datasets.

### 1.1 Related work

Cerebral microbleeds (CMB) are small, hypointense lesions appearing on gradient echo (GRE) or susceptibility-weighted (SWI) MRI scans. They are indicative of various cerebrovascular conditions, including small vessel disease, cerebral amyloid angiopathy, and Alzheimer’s disease. Accurate detection and quantification of CMB are crucial for diagnosis, prognosis, and monitoring. Traditional methods rely on visual inspection by trained radiologists, which is time-consuming and prone to inter-observer variability (Obuchowicz et al., 2020, Forsting et al., 2021). This has driven interest in automated methods, particularly those using deep learning. The detection of CMB is especially challenging due to their small size, low prevalence, and mimics such as calcifications and vascular anomalies. Early efforts using classical techniques included thresholding, region growing, radial symmetry transforms, and Support Vector Machines (SVM) (Ferlin et al., 2023, Kuijf et al., 2012, Bian et al., 2013, Morrison et al., 2018, Ateeq et al., 2023). Despite reducing some manual effort, these methods often suffered from high false-positive rates and poor generalisability. For example, Chesebro et al. (Chesebro et al., 2021) combined 2D gradients, Canny edge detection, and circular Hough transforms for candidate detection, followed by Frangi-filter-based blob analysis to reduce false positives. Still, the method resulted in nearly 20 false positives per case for GRE scans and 10 for SWI scans. To address these limitations, researchers increasingly turned to deep learning. CNN-based approaches, such as DEEPMIR (Rashid et al., 2021), trained models to differentiate between CMBs and iron deposits. Al-Masni et al. (Al-Masni et al., 2020) developed a multi-stage framework that integrates YOLO and CNNs to enhance robustness. Dadar et al. (Dadar et al., 2022) explored transfer learning for automated segmentation, while Suwalska et al. (Suwalska et al., 2022) proposed CMB-HUNT, a deep network specifically for CMB detection. Similarly, Kim et al. (Kim et al., 2025) developed a localisation-aware deep learning model for accurate anatomical mapping of CMBs. 3D CNNs have become popular due to their ability to incorporate volumetric context. For example, Chen et al. (Chen et al., 2019) applied a 3D deep residual network to detect radiation-induced CMBs. Tsuchida et al. (Tsuchida et al., 2024) proposed SHIVA-CMB, a robust tool trained on multi-source T2*GRE and SWI data. Khaffafi et al. (Khaffafi et al., 2025) leveraged multi-channel, multi-scale 3D CNNs to improve sensitivity and generalisability. Nishioka et al. (Nishioka et al., 2025) combined morphology filter banks with CNNs for robust detection

Knowledge distillation (Sundaresan et al., 2023) and ensemble detection (Lee et al., 2023) have also been applied to boost performance. Some studies have integrated image-level and contextual information, such as Ali et al. (Ali et al., 2023), who used IoMT frameworks, and Chen et al. (Chen et al., 2024), who incorporated transformer-based models. Sundaresan et al. (Sundaresan et al., 2025) also examined the spatial distribution and size of CMBs for characterisation. Synthetic data has proven valuable for training. Momeni et al. (Momeni et al., 2021, Momeni et al., 2021) generated synthetic CMBs using Gaussian models and GANs to augment datasets. To validate models on limited SWI data, some approaches, such as our pre-trained models on GRE (e.g., ADNI), are then fine-tuned using synthetic SWI data. Transfer learning has also been effective. Wang et al. (Wang et al., 2019) proposed a DenseNet-based framework, whereas Hong et al. (Hong et al., 2019) utilised a pre-trained ResNet-50. In summary, deep learning methods—especially 3D CNNs, ensemble techniques, and transfer learning—have significantly improved CMB detection accuracy and robustness. However, challenges such as high false favourable rates and modality variation remain. Our work addresses these by combining tailored patch extraction and 3D CNN classification, with a YOLO-based false positive reduction strategy.

## 2 Method

**Fig. 1:**
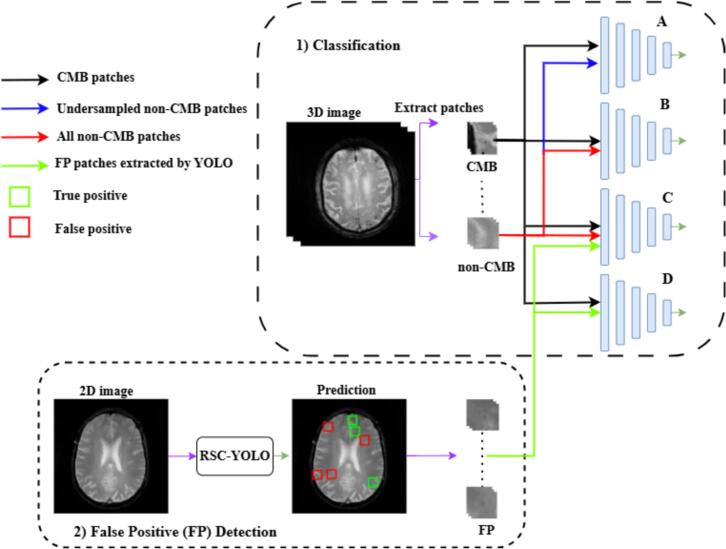
Overview of the proposed model. The framework comprises a 3D CNN for CMB classification and an RCS-YOLO model for false-positive detection. Four training strategies were explored for the 3D CNN: (A) training with all CMB patches and undersampled non-CMB patches; (B) training with all CMB patches and all non-CMB patches; (C) training with all CMB patches, all non-CMB patches, and false positive (FP) patches detected by YOLO; and (D) training with all CMB patches and YOLO-detected FP patches only.

This section outlines the proposed deep learning-based method for detecting cerebral microbleeds (CMBs), as illustrated in Fig. 1. Our approach is based on a patch-based 3D convolutional neural network (CNN), which we trained under several configurations to identify the most effective setup for this task.

Initially, 3D patches are extracted from the input MRI scans (details provided in Section 3.4.1). The 3D CNN is then trained using four different strategies: **A)** CMB patches combined with *undersampled* non-CMB patches. **B)** CMB patches combined with *all* non-CMB patches. **C)** CMB patches plus all non-CMB patches and *false positives (FPs)* detected by the RCS-YOLO model (see Section 3.4.3). **D)** CMB patches plus only the FPs detected by YOLO.

To improve model robustness, particularly in distinguishing CMBs from mimics, we utilised RCS-YOLO to detect challenging false positives that resemble real CMBs. These FP patches were then included in the training data to enhance generalisation and reduce false detections in real-world scenarios.

Key components of the CNN include convolutional layers, which are multiple convolutional layers used to learn spatial hierarchies of features from the input patches; pooling layers, which reduce the spatial dimensions and retain essential features; and fully connected layers, which are dense layers that integrate the extracted features and perform classification. As shown in Table 2, initially, two sets of Conv + BN + ReLU (Convolutional layer, Batch Normalisation, and ReLU activation function) layers with 32 filters of size 3 × 3 × 3 were used, followed by a max-pooling layer with a 2 × 2 × 2 window and a dropout layer with a rate of 0.3. This pattern was repeated with 64 and 128 filters, with the dropout rate increasing to 0.4 in the latter stage. Finally, fully connected linear layers were introduced, culminating in an output layer with two units. The architecture is designed to efficiently capture and process the intricate features of CMB while maintaining robustness against overfitting; see Table 3 for details of the network architecture.

**Table 2:** Proposed network architecture.

Layer Type	Filter Size	Rate
Conv + BN + Relu	32 × 3 × 3 × 3	−
Conv + BN + Relu	32 × 3 × 3 × 3	−
Maxpooling	2 × 2 × 2	−
Dropout	−	0.3
Conv + BN + Relu	64 × 3 × 3 × 3	−
Conv + BN + Relu	64 × 3 × 3 × 3	−
Maxpooling	2 × 2 × 2	−
Dropout	−	0.3
Conv + BN + Relu	128 × 3 × 3 × 3	−
Conv + BN + Relu	128 × 3 × 3 × 3	−
Maxpooling	2 × 2 × 2	−
Dropout	−	0.4
Linear	256	−
Dropout	−	0.4
Linear	2	−

**Table 3:** Number of patients, MRI, and CMB in training, validation, and test sets.

Dataset	Split	Patients	MRI	CMB
ADNI-GO	Train	28	73	142
	Val	7	9	53
ADNI-2	Train	287	955	3372
	Val	30	101	341
	Test	20	68	232
ADNI-3	Train	241	489	2485
	Val	30	60	158
	Test	20	34	87

## 3 Results

### 3.1 Dataset

**Fig. 2:**
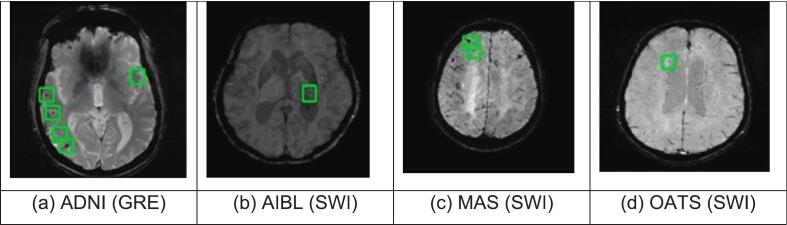
Sample images from each dataset with CMB highlighted using a green bounding box. (For interpretation of the references to colour in this figure legend, the reader is referred to the web version of this article.)

This work utilised two publicly available datasets (ADNI and AIBL) and two private datasets (MAS and OATS) to validate the proposed methodology. Fig. 2 shows a sample image from each dataset. Detailed information on each dataset is provided in the subsections.

#### 3.1.1 ADNI

The Alzheimer’s Disease Neuroimaging Initiative (ADNI) is a landmark research effort initiated in 2004 to investigate Alzheimer’s disease (AD) by collecting and analysing a wide range of clinical imaging (MRI and PET scans) and genetic and biochemical biomarkers. The project aims to develop and validate biomarkers for the early detection and tracking of AD progression.

The ADNI dataset is an extensive resource that spans multiple study phases (ADNI-1, ADNIGO, ADNI-2, ADNI-3, and ADNI-4). Its diversity is a significant asset for researchers examining the mechanisms, progression and potential treatments for AD. The broad scope of the ADNI dataset makes it invaluable for advancing the understanding and management of AD.

In addition, the Mayo Clinic has provided detailed data on the number and location of all CMB and superficial siderosis in T2 GRE images through their IDA database for several patients in ADNIGO, ADNI-2 and ADNI-3 phases. The CMB is assessed in anatomical space, and right anterior and superior anatomical coordinates (RAS locations) for CMB are provided. Table 1 presents the number of patients, MRI scans and CMB for patients with definite CMB.

**Table 1:** Number of patients, GRE MRI, and CMB in ADNI-GO, ADNI-2, and ADNI-3 datasets. RAS refers to the Right-Anterior-Superior coordinate system, and XYZ represents voxel coordinates in 3D space (X: left–right, Y: posterior-anterior, Z: inferior-superior).

Phase	# Patients		#MRIs		#CMB	
	#RAS	#XYZ	#RAS	#XYZ	#RAS	#XYZ
ADNI-GO	35	35	82	82	195	195
ADNI-2	337	334	1133	1117	8432	3343
ADNI-3	297	297	586	586	2733	2733

Each patient undergoes one or more MRI scans per phase, while some participate in multiple phases. Among the reported patients in Table 1, nine participated in all three phases, i.e., ADNI-GO, ADNI-2, and ADNI-3.

The variability in the number of CMBs in this dataset is relatively high. There are patients with just one CMB and more than 500 CMBs.

As mentioned, CMB in the ADNI dataset was assessed in anatomical space (RAS), and the images were neither standardised nor registered. To ensure accurate automated analysis, it was necessary to convert all RAS locations into global X, Y, and Z coordinates. First, all DICOM files were converted to NIfTI format using the MRIcroGL software (Suwalska et al., 2022). Next, FSLeyes (Kim et al., 2025) was employed to translate the RAS locations into XYZ coordinates. Table 1 summarises the conversion results, showing that the RAS locations of all CMB in ADNI-GO and ADNI-3 were successfully transformed into XYZ coordinates. Due to the time-consuming nature of converting RAS locations, we excluded 13 patients in the ADNI-2 dataset with a very high burden of CMB to streamline the analysis process.

To validate the robustness of the proposed approach, we extracted 439 MRI scans (121 from ADNI-GO, 202 from ADNI-2 and 116 from ADNI-3) corresponding to 165 patients (49 from ADNI-GO, 50 from ADNI-2 and 66 from ADNI-3). These scans were sourced from normal cases within the ADNI dataset.

#### 3.1.2 AIBL

The Australian Imaging, Biomarkers and Lifestyle (AIBL) (Ellis et al., 2009) dataset is a comprehensive collection of neuroimaging, clinical, genetic and lifestyle data primarily aimed at understanding AD and its progression. It includes both cross-sectional and longitudinal data from normal individuals, mild cognitive impairment (MCI) patients, and those diagnosed with AD. The dataset comprises brain imaging data, including MRI and PET scans, cognitive assessments, and biomarker data (e.g., amyloid and tau). One notable contribution to the AIBL dataset is the work by Momeni et al. (Momeni et al., 2021), who labelled and published 57 SWI MRIs and 146 CMB locations. They also proposed a synthetic method for generating CMB, creating 10 different versions for each of the 313 normal SWI MRIs, thereby generating a total of 3,130 MRIs, including 31,300 synthetic CMBs. In this work, we utilised 57 SWI MRIs from patients with confirmed CMB and 3,130 MRIs containing synthetically generated CMB.

#### 3.1.3 MAS and OATS

For the evaluation of the proposed method, two private datasets — the MAS (Sydney Memory and Ageing Study) (Sachdev et al., 2010) and the OATS (Older Australian Twins Study) (Sachdev et al., 2013) — from the Centre for Healthy Brain Ageing (CHeBA) were utilised.MAS is a longitudinal study initiated in 1996, focusing on cognitive ageing in community-dwelling adults aged 70 and above. MRI scans in the MAS dataset were acquired using a Philips 3 T Achieva Quasar Dual scanner with the following SWI parameters: repetition time (TR) = 25.33 ms, echo time (TE) = 40.33 ms, slice thickness = 1.1 mm, and field of view (FOV) = 240 × 132 × 215 mm^3^ with 0.55 mm slice overlap.

OATS, launched in 2007, complements MAS by studying monozygotic and dizygotic twins aged 65 and above, aiming to disentangle genetic and environmental factors in brain ageing. MRI scans were performed on a 1.5 T Siemens MAGNETOM Avanto scanner using a 3D gradient-echo SWI sequence: TR = 38 ms, TE = 30 ms, flip angle = 15°, FOV = 240 × 240 mm^2^, matrix size = 258 × 320, and slice thickness = 1.5 mm.

Both studies combine neuroimaging, genetic, and clinical data to investigate cognitive decline and neurodegeneration. SWI MRIs in both datasets enabled detailed analysis of cerebral microbleeds (CMBs). A subset of the MAS dataset includes 30 MRIs with 60 labelled CMBs, while the OATS subset contains 23 MRIs with 54 labelled CMBs.

### 3.2 Data pre-processing

Since the CMBs are small and, in most cases, quite rare (occurring one or two times per MRI), using the entire image is overkill and will only slow down the processing. Therefore, in this work, we used patch-based 3D volumes for processing. After conducting comprehensive preliminary experiments, we found that an 11 × 11 × 11 (pixels × pixels × slices) configuration performed best. For model training, validation, and testing, the ADNIGO, ADNI-2, and ADNI-3 datasets were divided into distinct sets, as illustrated in Table 3. This initial stage of dataset preparation ensures a balanced distribution across different study phases, facilitating robust model evaluation and performance assessment. The organised grouping allows for practical training while maintaining data integrity and consistency across various sets. It is worth noting that all patients in the validation and test sets just attended one phase of ADNI. A patient-level split between training, validation and test sets was adopted to prevent data leakage and maintain subject-level independence. We also used zero mean and unique variance to normalise all the images in all datasets.

### 3.3 Implementation

In our implementation, we used the ADAM optimiser with a learning rate (lr) of 0.01 and a weight decay of 0.0001 to apply L2 regularisation and prevent overfitting. The model was trained using 200 epochs with a batch size of 128 and optimised using the CrossEntropyLoss function for binary classification. Data augmentation was applied using TorchIO (Perez-Garcia et al., 2021), and the final training dataset was constructed by concatenating the original and augmented samples. The training, validation, and testing were conducted using PyTorch 1.13.1 (Paszke et al., 2019), utilising 1 to 8 CPU(Intel(R) Xeon(R) Gold 6248R) threads via PyTorch DataLoaders with appropriate shuffling and parallel data loading. We applied various augmentation methods, including flipping (vertical and horizontal), rescaling, and intensity changes. To evaluate model performance, we used Balanced accuracy (Al-Masni et al., 2020); Area Under the Curve (AUC) (Sundaresan et al., 2023), Precision (Cummings et al., 2023), Sensitivity (Roytman et al., 2023), and F1-score (Chesebro et al., 2021), as shown below.

(1) $$\mathrm{Balancedaccuracy}=\frac{1}{2}\left(\frac{\mathrm{TP}}{\mathrm{TP}+FN}+\frac{\mathrm{TN}}{\mathrm{TN}+FP}\right)$$

(2) $$\mathrm{Precision}=\frac{\mathrm{TP}}{\mathrm{TP}+FP}$$

(3) $$\mathrm{Sensitivity}=\frac{\mathrm{TP}}{\mathrm{TP}+FN}$$

(4) $$F1-Score=2\times \frac{\mathrm{Precision}\times Sensitivity}{\mathrm{Precision}\times Sensitivity}$$

### 3.4 Experimental results on ADNI

#### 3.4.1 Patch extraction and selection

We extracted 3D patches using two distinct methods. First, we employed a non-overlapping sliding window approach, scanning from left to right and top to bottom to extract patches (called sliding windows). A patch containing a CMB is labelled as CMB; otherwise, it is labelled as non-CMB.

Second, we specifically extracted patches where the CMB is centred within the patch to ensure it occupies the focal point (called centralised CMB). Non-CMB patches were extracted using the same sliding window approach as before. This method enables the network to leverage spatial information from neighbouring slices, thereby improving its ability to detect the CMB effectively.

Given the rarity of CMB, there is a significant imbalance between the number of CMB and non-CMB patches. To address this issue, the most commonly used approach in the literature is undersampling, which reduces the number of non-CMB patches to mitigate the imbalance (Wang et al., 2019, Sa-ngiem et al., 2019, Wang et al., 2020). In this section, we also applied undersampling to reduce the number of non-CMB patches.

As shown in Table 4, the centralised CMB approach achieved better results (Balanced accuracy: 0.953, AUC: 0.955, Precision: 0.954, Sensitivity: 0.92 and F1-score: 0.93). Centring the CMB within the patch allows the network to capture spatial information from neighbouring slices effectively. In contrast, the sliding window approach may locate the CMB near the edge or corner of a patch, limiting the amount of contextual information available and thereby reducing model performance.

**Table 4:** Performance comparison of models trained on sliding window and CMB-centred patches extracted from ADNI. The models utilised all available CMB patches together with undersampled non-CMB patches.

Model	Balanced Accuracy	AUC	Precision	Sensitivity	F1-score
Sliding window	0.821	0.853	0.725	0.896	0.818
Centralized CMB	0.953	0.955	0.954	0.92	0.93

We adopted the centralised CMB method for patch extraction for the remainder of the paper based on the results obtained.

We also investigated the generalisation performance of the model trained with undersampling of normal patches. While undersampling is a widely adopted method in the literature, it raises a critical question: Does this approach yield realistic results? Specifically, we explored two key scenarios: (1) training the model using undersampled patches but evaluating it on all image patches, and (2) training the model using all patches without undersampling. The critical question is addressed in Table 5, which highlights the impact of the different approaches on model performance. As shown in Table 5, testing the model with undersampled training on all patches in the test set resulted in a significant drop in performance. Specifically, the Area Under the Curve (AUC) decreased by 0.012 per cent, Precision dropped by 0.626 per cent, Sensitivity fell by 0.023 per cent, and the F1-score reduced by 0.491 per cent compared to the model trained and tested on the under-sampled images, primarily due to an increase in false positives (FP). This highlights that undersampling is not a reliable approach for identifying CMB in the complete set of patches. In contrast, training and testing the model using all available patches (Exhaustive Training and Testing) led to a substantial performance improvement compared to undersampled training with exhaustive testing. This enhancement can be attributed to the network being exposed to a broader range of CMB mimics during training, thereby improving its ability to accurately detect CMB in the test set while reducing false positives.

**Table 5:** Comparison of CMB detection model generalisability on ADNI: Trained with and without undersampling. Category 1 (fewer than four CMBs, 86 scans), 2 (five to nine CMBs, 10 scans), 3 (ten or more CMBs, six scans). Evaluation metrics: False Positive (FP), True Positive (TP), False Negative (FN), and True Negative (TN).

Model	Category	Balanced Accuracy	AUC	Precision	Sensitivity	F1-score	FP	TP	FN	TN
Undersampled Training and Testing	Entire Test set	0.953	0.955	0.954	0.92	0.93	16	292	27	1579
	Category 1	0.945	0.945	0.944	0.9	0.921	7	118	13	648
	Category 2	0.971	0.97	0.981	0.945	0.962	1	52	3	274
	Category 3	0.953	0.952	0.938	0.917	0.927	8	122	11	657
Undersampled Training with Exhaustive Testing	Entire Test set	0.942	0.943	0.328	0.897	0.439	1187	284	35	166,345
	Category 1	0.928	0.928	0.105	0.862	0.187	960	113	18	148,213
	Category 2	0.957	0.956	0.242	0.927	0.384	159	51	4	11,524
	Category 3	0.946	0.946	0.638	0.902	0.747	68	120	13	6608
Exhaustive Training and Testing	Entire Test set	0.893	0.9	0.891	0.786	0.834	28	251	68	167,504
	Category 1	0.84	0.889	0.831	0.679	0.747	18	89	42	149,155
	Category 2	0.927	0.893	0.87	0.854	0.861	7	47	8	11,676
	Category 3	0.913	0.92	0.973	0.827	0.894	3	110	23	6673

Additionally, the number of CMBs is crucial in determining treatment options (Cummings et al., 2023). To evaluate the robustness of the trained models, we assessed their performance on the entire test set as well as within specific subgroups: Category 1 (patients with fewer than five CMBs), Category 2 (patients with five to nine CMBs) and Category 3 (patients with ten or more CMBs). This stratified evaluation enables us to examine the effectiveness of the models at varying levels of CMB severity, ensuring reliable performance across different patient groups. In the test set, Category 1 consists of 86 MRIs with 131 CMBs, Category 2 includes 10 MRIs with 55 CMBs, and Category 3 contains 6 MRIs with 133 CMBs.

As shown in Table 5, the network performance (Balanced accuracy, AUC, Precision, Sensitivity and F1-score) of the Undersampled Training and Testing model is quite good across all three categories. In the second-best model (Exhaustive Training and Testing), the third category achieves the best AUC, Precision, and F1-score. Overall, since the proposed method is patch-based and decisions are made solely based on individual patches rather than the severity of the patient’s condition, there are no significant differences in performance across the three categories.

#### 3.4.2 Comparison with other models

We now compare the performance of the proposed CNN for CMB detection with that of EfficientNet (Tan and Le, 2019), ResNet (Zifeng et al., 2019), and DenseNet (Huang et al., 2017), which are state-of-the-art classification networks. As summarised in Table 6, the proposed CNN outperformed EfficientNet3D-Tiny, 3D DenseNet-15, and ResNet10 on the ADNI test set when trained and tested using all slices of the selected ADNI database, focusing on Precision and F1-score metrics.

**Table 6:** Comparison of the proposed model with EfficientNet, Densenet, and Resnet for ADNI, using all slices for the training and testing.

Model	Balanced accuracy	AUC	Precision	Sensitivity	F1	FP	TP	FN	TN
EfficientNet3D-Tiny	0.904	0.904	0.047	0.84	0.089	5370	268	51	162,162
3D DenseNet-15	0.871	0.871	0.877	0.742	0.804	33	237	82	167,499
**Resnet10**	0.826	0.825	0.832	0.652	0.731	42	208	111	167,490
**Proposed model**	0.893	0.893	0.899	0.786	0.839	28	251	68	167,504

Although EfficientNet3D-Tiny achieved the highest true positive (TP) rate of 268, it also produced an excessively high false positive (FP) rate of 5370. In contrast, the proposed CNN achieved the lowest FP count (28) while maintaining a high TP rate of 251. This performance highlights the robustness of the proposed model, particularly given the limited training data size. 3D DenseNet-15 delivered the second-best results, demonstrating a balance between TP and FP rates, but still fell short compared to the proposed model.

#### 3.4.3 Improving robustness of the proposed model

As demonstrated so far, a sliding window approach was used to select non-CMB patches, and the results highlighted the impact of non-CMB patch selection on the performance of the trained model. Instead of using all the sliding window-extracted patches or selecting a random subset, we propose leveraging the YOLO model for non-CMB patch extraction. This approach aims to identify realistic CMB-mimicking patches, thereby improving the Robustness of the proposed model.

**Fig. 3:**
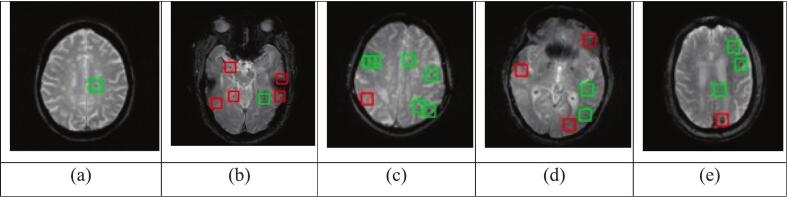
Sample predictions by the YOLO non-CMB detection model, highlighting true positives (green) and false positives (red) in the detection results (ADNI dataset). (For interpretation of the references to colour in this figure legend, the reader is referred to the web version of this article.)

After conducting preliminary experiments, we found that the RCS-YOLO network (Kang et al., 2023), which was developed for brain tumour detection, produced superior results. A CMB detection model was trained on the training set and tested on 2D images from the test set in the ADNI dataset (the same split that has been used for classification). The implementation strictly adhered to the original paper, using the specified parameters for training. The accuracy of the RCS-YOLO model was relatively low, achieving 0.74 on the training set, 0.82 on the validation set and 0.53 on the test set. This is likely due to the limited size of the training dataset and the 2D architecture employed, which does not utilise complete spatial information for detecting CMB. However, as shown in Fig. 3, the false positives identified by the model closely resemble the actual CMB. Incorporating these patches into the CNN training set for CMB classification has the potential to enhance model robustness and overall performance.

The YOLO CMB detection model identified 4,900 false positives, comprising 4,268 on the training set, 394 on the validation and 238 on the test set. In this section, we evaluate the performance and reliability of the proposed CNN model using only the non-CMB patches (false positives detected by YOLO) and CMB patches. To ensure a comprehensive assessment, five-fold cross-validation was employed five times (see Table 7).

**Table 7:** Results of the proposed model were evaluated through five-fold cross-validation on the ADNI dataset. The training and test datasets consisted of CMB patches and false-positive patches extracted using YOLO.

Repeat	Balanced accuracy	AUC	Precision	Sensitivity	F1-score	FP	TP	FN	TN
R1	0.875	0.874	0.887	0.910	0.898	157.4	1249.2	121.8	822.6
R2	0.880	0.879	0.891	0.916	0.903	153.2	1256.8	114.2	826.8
R3	0.874	0.873	0.880	0.922	0.900	171.6	1265.4	105.6	808.4
R4	0.888	0.888	0.901	0.916	0.908	137.2	1256.8	114.2	842.8
R5	0.877	0.876	0.884	0.921	0.902	164.4	1264	107	815.6
Avg	0.879	0.878	0.888	0.917	0.902	156.76	1258.44	112.56	823.24
std	0.002	0.005	0.007	0.004	0.003	12.983	6.521	6.521	12.983

As shown in Table 7, after performing five times five-fold cross-validation, the proposed model improved the Sensitivity by 0.131 and the F1-score by 0.063 compared to using only all the sliding window slices as non-CMB patches.

#### 3.4.4 CMB prediction on normal cases

**Fig. 4:**
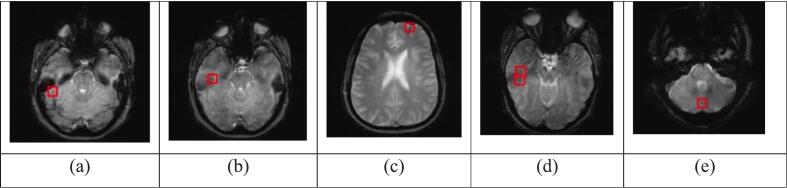
Samples of FP detected by YOLO on normal cases (ADNI dataset).

To evaluate the robustness of the proposed model, we used the trained YOLO model to predict CMB in normal (control) cases. As previously outlined, 439 MRI scans were extracted from ADNI-GO, ADNI-2 and ADNI-3. The trained YOLO model was used to predict CMB in the normal cases from the ADNI dataset. Five hundred sixty-six false-positive CMBs were detected across the 439 MRI scans, averaging 1.2 false positives per MRI scan. A sample of the false positives detected by YOLO in the normal cases is shown in Fig. 4.

As shown in Fig. 4, the false positives detected in normal cases closely resemble true CMB.

We designed a stepwise training strategy to empirically assess how different types of non-CMB patches influence model robustness and false positive reduction. The configurations were designed progressively, starting with standard CMB patches and randomly sampled non-CMB patches using a sliding window. This established a baseline for model performance.

In the second configuration, we replaced the random non-CMB patches with more realistic mimics identified by YOLO, which more closely resemble true CMBs. The third and fourth configurations combined both types of non-CMB patches, ie sliding window and YOLO-detected false positives, to evaluate whether a comprehensive and diverse training set would further enhance generalisation and reduce false positives.

This sequence was designed empirically based on prior findings in the literature and our pilot experiments. While not hypothesis-driven in a strict sense, this design effectively demonstrates, through incremental evaluation, how each type of non-CMB data contributes to improving the model. A more systematic ablation study will be considered in future work.

We implemented a series of training strategies to reduce the number of false positives in these normal cases. These strategies were designed to improve model performance by incorporating different types of patches:

1. Training with CMB and non-CMB patches extracted by a sliding window: This method involves using standard CMB patches combined with randomly selected non-CMB patches extracted using a sliding window technique. This approach reduced the number of false positives to 349
2. Training with CMB and Non-CMB patches extracted by YOLO: In this strategy, we utilised non-CMB patches identified by YOLO, which more accurately mimic real CMB compared to randomly selected patches from the sliding window. This reduced the false positives to 201
3. Training with CMB patches and selected non-CMB patches (sliding window) and FP patches (YOLO): This approach incorporated both standard CMB patches and randomly selected non-CMB patches, in addition to false positive patches detected by YOLO. This further reduced the false positives to 157
4. Training with CMB patches, non-CMB patches (sliding window), and FP patches (YOLO): This method combines CMB patches, non-CMB patches from the sliding window, and false positive patches detected by YOLO to provide a comprehensive training set. This strategy led to a further reduction in false positives to 132

These results demonstrate that training the model using CMB patches in conjunction with all non-CMB patches extracted by the sliding window, as well as false positive patches detected by YOLO, creates a more robust model. This approach effectively reduces false positives in normal cases, which is crucial to achieving more accurate diagnoses and informing treatment plans.

### 3.5 Experimental results on AIBL dataset

Based on the results obtained from the ADNI dataset, which demonstrate the effectiveness of false positive (FP) patches extracted by YOLO, and considering the relatively small size of the AIBL dataset, which contains only 146 CMBs, we implemented three strategies to train the YOLO model to extract more realistic mimics of non-CMB patches:

1. Training with all image slices of synthetic SWI images: In this strategy, YOLO was trained using the entire set of synthetic SWI image slices, including both CMB and non-CMB slices
2. Training with synthetic SWI slices containing only synthetic CMB: This approach focused on training YOLO using only slices from the SWI dataset that contained synthetic CMB, excluding those without CMB
3. Pretraining on ADNI and fine-tuning on synthetic CMB slices: The YOLO model was first pre-trained on the ADNI dataset and then fine-tuned using only the slices containing synthetic CMB from the SWI dataset

Among these strategies, the third approach — pretraining on ADNI followed by fine-tuning on synthetic CMB slices — achieved the best performance in both accuracy and false-positive reduction. Using this approach, the YOLO model detected 2,811 false positives across 57 MRIs (4,560 slices), averaging approximately 1.6 false positives per slice.

After extracting non-CMB patches using the YOLO model, the proposed CNN model was trained with both CMB and non-CMB patches. Five-fold cross-validation was performed and repeated five times to validate the model. The results are presented in Table 8. The proposed model achieved excellent performance on the 57 SWI MRI scans from the AIBL dataset, with a balanced accuracy of 0.968, AUC of 0.957, Precision of 0.956, Sensitivity of 0.938, and an F1-score of 0.946. After applying five-fold cross-validation, the model achieved an average of 1.2 false positives (FP) across all 4,560 slices from the 57 MRIs. In comparison, Momeni et al. (Momeni et al., 2021) utilised synthetic data to train a Random Forest classifier and reported an average of 9 false positives per scan.

**Table 8:** Results of the proposed model were evaluated through five-fold cross-validation on AIBL. The training and test datasets consist of CMB patches and false positive patches extracted by YOLO from AIBL.

Repeat	Balanced accuracy	AUC	Precision	Sensitivity	F1-score	FP	TP	FN	TN
R1	0.964	0.964	0.964	0.931	0.947	1	27.2	2	561.2
R2	0.964	0.964	0.965	0.931	0.947	1	27.2	2	561.2
R3	0.974	0.929	0.929	0.951	0.939	2.2	27.8	1.4	560
R4	0.968	0.958	0.958	0.938	0.947	1.2	27.4	1.8	561
R5	0.968	0.966	0.966	0.938	0.95	1	27.4	1.8	561.2
Avg	0.968	0.956	0.956	0.938	0.946	1.28	27.4	1.8	560.92
Std	0.002	0.015	0.015	0.008	0.004	0.521	0.244	0.244	0.521

**Fig. 5:**
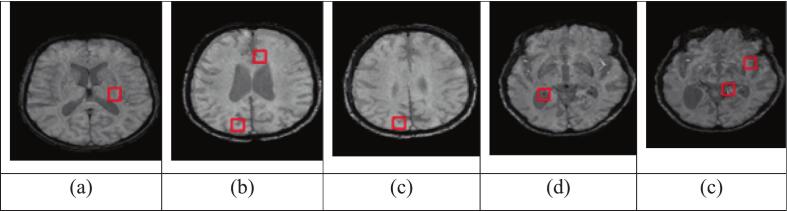
samples of FP detected by YOLO on normal cases (AIBL dataset).

To further evaluate the robustness of the proposed model, we used 131 SWI MRIs of normal cases for CMB recognition. YOLO was pre-trained on the ADNI dataset and fine-tuned with synthetic CMB data; the resulting model was used to predict CMB in 313 SWI MRIs from normal cases comprising 25,040 scans. YOLO detected 14,030 false positives (FP), corresponding to an average of 44.8 FP per MRI or approximately 0.57 FP per slice. Samples of the false positives detected in the normal cases are presented in Fig. 5.

To further reduce the false positives in normal cases, we trained the CNN recognition model on both CMB patches and non-CMB patches identified by YOLO. Predictions were then made on the false positive patches extracted by YOLO from 313 normal cases. After five repetitions of fivefold cross-validation, the average number of false positives was reduced to 92.3, demonstrating the model’s effectiveness in minimising false positives.

### 3.6 Experimental results on MAS and OATS datasets

Two radiologists with expertise in CMB detection independently reviewed the MAS and OATS datasets to identify CMB. Following their annotations, 54 CMBs were identified across 24 SWI MRIs (1656 slices) in the OATS dataset, and 60 CMBs were identified across 30 SWI MRIs (7200 slices) in the MAS dataset.

Since both datasets are relatively small, they are insufficient for directly training the YOLO model. Therefore, we utilised a pre-trained YOLO model (trained on the ADNI dataset and finetuned on synthetic CMBs) to extract false positive (FP) patches.

On the OATS dataset, the model detected 1,579 false positives (FPs) from 1,656 slices, averaging 95 FPs per MRI and 0.95 FPs per slice. Similarly, on the MAS dataset, 7,875 FP were identified across 7,200 slices, resulting in an average of 131.25 FP per MRI and 0.91 FP per slice. These elevated FP rates were expected, as the YOLO model was pre-trained on GRE MRIs from the ADNI dataset and fine-tuned using synthetic CMB derived from SWI data without exposure to real CMB during training. Additionally, the inherently noisier nature of SWI MRIs contributes to the higher FP rates compared to GRE images. Specifically, the MAS dataset contains more MRIs with severe artefacts, further explaining the elevated FP count observed in this dataset.

To reduce the false positives (FP) in both datasets, we trained the proposed CNN using a fivefold cross-validation strategy to ensure robust evaluation. Initially, the data was partitioned into five folds, and the number of true positives (TPs) was evenly distributed across all folds.

Since the number of CMB patches is minimal and the training data is highly imbalanced, fivefold cross-validation provides a more robust validation technique. Table 9 presents the results obtained after performing five repetitions of five-fold cross-validation on the OATS dataset. The table shows that the model achieved high Balanced accuracy, AUC, Precision, Sensitivity, and F1-score.

**Table 9:** The results of the proposed CNN model were evaluated through a five-fold cross-validation. The training and test datasets consist of CMB patches and false positive patches extracted by YOLO from the OATS dataset.

Repeat	Balanced Accuracy	AUC	Precision	Sensitivity	F1-score	FP	TP	FN	TN
R1	0.936	0.935	0.98	0.872	0.921	0.2	9.4	1.4	315.6
R2	0.924	0.924	0.941	0.850	0.892	0.6	9.2	1.6	315.2
R3	0.934	0.933	0.960	0.869	0.908	0.4	9.4	1.4	315.4
R4	0.906	0.905	0.918	0.814	0.858	0.8	8.8	2	315
R5	0.953	0.952	0.945	0.907	0.921	0.6	9.8	1	314.2
Avg	0.93	0.930	0.949	0.862	0.900	0.52	9.32	1.48	315.28
Std	0.012	0.017	0.023	0.033	0.026	0.228	0.363	0.363	0.228

Despite the limited number of CMB and the highly imbalanced nature of the data, the proposed model demonstrated commendable performance.

Table 10 presents the results of five-fold cross-validation repeated five times on the MAS dataset. The MAS dataset’s performance was lower than that of the OATS dataset, which can be attributed to the higher number of false positives detected in the MAS dataset. Nonetheless, the proposed model still achieved acceptable performance.

**Table 10:** The results of the proposed model were evaluated using five-fold cross-validation. The training and test datasets consist of CMB patches and false positive patches extracted by YOLO from the MAS dataset.

Repeat	Balanced accuracy	AUC	Precision	Sensitivity	F1-score	FP	TP	FN	TN
R1	0.899	0.899	0.946	0.799	0.861	0.6	9.6	2.4	1574.4
R2	0.866	0.866	0.94	0.733	0.822	0.6	8.8	3.2	1574.4
R3	0.875	0.874	0.977	0.749	0.847	0.2	9	3	1574.8
R4	0.883	0.882	0.931	0.766	0.833	0.8	9.2	2.8	1574.2
R5	0.924	0.924	0.945	0.849	0.892	0.6	10.2	1.8	1574.4
Avg	0.889	0.889	0.948	0.779	0.851	0.56	9.36	2.64	1574.44
Std	0.017	0.023	0.017	0.046	0.027	0.219	0.554	0.554	0.219

## 4 Discussion

This paper presents a robust deep learning-based framework for recognising cerebral microbleeds (CMB) in GRE and SWI MRIs. The model was validated using four datasets: two public datasets (ADNI [GRE] and AIBL [SWI]) and two private datasets (OATS [SWI] and MAS [SWI]). Recognising CMB is inherently challenging even for experienced radiologists due to its rarity, small size, and resemblance to other features. Moreover, machine learning and deep learning models often struggle with imbalanced datasets and the high incidence of false positives (FP), which are common challenges in the field.

To address these limitations, we proposed a 3D patch-based CNN for detecting CMB patches. A key challenge was managing the class imbalance in the datasets. While undersampling is widely used to handle imbalance, it often compromises model generalisability and robustness, particularly in normal cases, and increases the risk of misdiagnoses. To overcome this, we employed a YOLO model to detect FP patches, which better resemble real CMB than randomly sampled non-CMB patches. This strategy significantly enhanced the model's robustness and effectiveness.

**Validation on Public Datasets:** On the ADNI dataset with 6,870 CMB, the proposed CNN model achieved strong performance after five-times five-fold cross-validation using CMB patches and FP patches detected by YOLO with a Sensitivity of 0.917 and an F1-score of 0.902. Additionally, we validated the model on normal cases using 439 MRIs from ADNI-GO, ADNI-2 and ADNI-3. By incorporating non-CMB patches extracted through a sliding window and FP patches detected by YOLO, the model achieved a remarkably low FP rate of only 132 FPs across all 439 MRIs.

On a subset of the AIBL dataset, we trained the YOLO model on the ADNI dataset and fine-tuned it on a synthetic CMB SWI dataset to extract non-CMB patches. The model achieved excellent results using five-times five-fold cross-validation with these patches: AUC = 0.967, Precision = 0.956, Sensitivity = 0.938, and F1-score = 0.946. When evaluated on 313 normal MRIs, the model maintained a low average FP rate of 92.3 % across all cases, demonstrating its robustness.

**Validation on Private Datasets:** For the smaller OATS and MAS datasets, which contain fewer CMBs, we also utilised the pre-trained YOLO model, trained on the ADNI dataset and fine-tuned on the synthetic CMB SWI dataset, to extract non-CMB patches. The proposed model achieved excellent results on the OATS dataset: AUC = 0.930, Precision = 0.949, Sensitivity = 0.862, and F1-score = 0.900. Similarly, the model performed well for the MAS dataset, with an AUC of 0.889, precision of 0.948, sensitivity of 0.779, and F1-score of 0.851.

**Comparison with other models:** Since there is no previous work on CMB in the ADNI dataset, we evaluated the performance of the proposed 3D CNN by comparing it with three well-known classification models: EfficientNet3D-Tiny (Tan and Le, 2019), ResNet10 (Zifeng et al., 2019) and 3D DenseNet-15 (Huang et al., 2017). Our proposed model achieved 0.011 lower AUC and 0.054 lower sensitivity compared to EfficientNet3D-Tiny; however, it outperformed EfficientNet3D-Tiny in terms of precision and F1 score, as EfficientNet3D-Tiny exhibited a significantly higher false positive rate. Further, compared to 3D DenseNet-15 and ResNet10, our model demonstrated superior performance across multiple metrics, including accuracy, AUC, precision, sensitivity and F1 score. Further, compared to related work on the AIBL dataset (Momeni et al., 2021), which reported nine false positives per scan, our approach successfully reduced this number to 1.2 after performing five-fold cross-validation five times.

**Novelty and clinical relevance:** To the best of the authors’ knowledge, this is the first study to utilise the ADNI dataset, which is one of the most extensive publicly available GRE datasets, for automatic CMB detection. As the dataset was not already preprocessed, extensive preprocessing was performed to prepare it for CMB detection. To validate our model, we also employed three additional SWI datasets, including both public and private sources.

A comprehensive set of experiments was conducted to address the issue of class imbalance in CMB detection. More importantly, to enhance model robustness and reduce false positives (FPs) in healthy cases, we leveraged a YOLO model to detect CMB mimics, treating them as false positives and incorporating them into the training set. This significantly reduced FP in both GRE and SWI data.

Furthermore, our experiments demonstrate that different training strategies have varying impacts on model performance, depending on the objective. If the goal is to maximise detection accuracy and AUC, undersampling can be a practical approach. However, to improve model robustness in distinguishing true CMB from mimics in normal cases, the use of CMB mimics in training is necessary. Finally, our findings indicate that the proposed framework performs effectively across both large and small datasets.

From a clinical perspective, accurate and automated CMB detection is crucial for early diagnosis and risk assessment of cerebrovascular diseases, including cerebral amyloid angiopathy and intracerebral hemorrhage. Reducing false positives is particularly important in clinical workflows to prevent unnecessary interventions and misdiagnoses. By improving the precision and robustness of CMB detection across different imaging modalities, our work has the potential to enhance decision-making in clinical practice and support neurologists in the assessment of patients with neurological disorders.

**Limitations and future works:** Despite our model demonstrating strong performance on CMB detection, the selection of CMB patches for training requires labelled data. As we utilised only one modality per dataset and a 2D YOLO model, the YOLO model performance was insufficient for accurate CMB patch detection. Therefore, we used its results solely to identify CMB mimics as false positives.

In our experiments, we also observed that the size and availability of training data significantly impacted the results. The SWI datasets (AIBL, OATS, and MAS) available to us were relatively small and therefore not sufficient to train the YOLO model directly. As a result, we pretrained YOLO on the ADNI dataset (GRE) and fine-tuned it using synthetic SWI CMB scans. This approach led to a higher rate of false positives when applied to real SWI CMB scans. Although many of these false positives were effectively filtered out in the second stage using the CNN, we believe that training YOLO directly on real SWI CMB data would substantially reduce the number of false positives and improve overall detection performance.

While our proposed two-stage framework, consisting of a 3D CNN for CMB classification and a YOLO-based module for false positive (FP) detection and candidate enrichment, demonstrated improved robustness and detection performance, it has limitations. First, YOLO was not used directly for CMB detection due to the limited size of the SWI datasets (AIBL, MAS, and OATS), which are insufficient to train a high-performing object detector. Instead, YOLO was primarily used to extract FP cases and potential mimics, enriching the CNN training set to improve the model's robustness for CMB detection on normal cases. This indirect usage limits the potential of end-to-end optimisation. Second, because the CNN and YOLO models are trained independently, their feature representations are not shared, and YOLO’s learned weights cannot be transferred to the CNN. This separation restricts the ability to fine-tune the entire pipeline jointly, potentially leading to suboptimal feature integration. On the other hand, end-to-end training requires much larger datasets. Future work will explore the use of larger, multimodal datasets and the design of unified architectures that allow joint training and better feature fusion across detection and classification stages.

It is worth noting that the primary goal of this study was to demonstrate the adaptability and robustness of the proposed framework across diverse datasets with varying imaging modalities (e.g., GRE and SWI) and acquisition parameters. Rather than developing a universally generalisable model trained once and applied to all datasets, we focused on evaluating how well the framework could be tailored to each dataset's characteristics through retraining. While this approach ensures high performance within each dataset, it does require retraining the 3D CNN for new domains, which may limit scalability in clinical practice. We acknowledge that this impacts generalisability and increases the deployment burden. To address this limitation, future work will focus on incorporating domain adaptation or transfer learning strategies that can minimise the need for retraining while maintaining performance across datasets.

As part of future work, we aim to implement a 3D YOLO model for CMB detection. By incorporating spatial information, we anticipate enhancing detection accuracy and reducing false positive rates, particularly in the early stages. The transition to a single-stage detection approach will improve efficiency while maintaining high performance. Furthermore, we plan to integrate localisation capabilities into the model, enabling precise identification of CMB locations, which is essential for treatment planning and improving clinical outcomes. Also, exploring how lesion size affects detection performance would be an interesting direction for future work.

## 4 Conclusion

Our experiments highlight the limitations of undersampling for imbalanced datasets, particularly for large datasets like ADNI, where the goal is to enhance model robustness and generalisability. By incorporating challenging FP patches detected by YOLO into the training process, our recognition model demonstrated improved performance and reduced FP rates even on smaller datasets such as AIBL, OATS and MAS.

An essential clinical consideration in CMB treatment planning is the number of CMBs present in a patient. Treatment is often reconsidered if the number of CMBs exceeds four. Our model, employing a two-stage approach with a patch-based CNN, performs detection on individual patches rather than the entire image. This approach ensures high detection performance across all CMB severity categories: Category 1 (Fewer than four CMB), Category 2 (five to nine CMB), and Category 3 (ten or more CMB). This study demonstrates that our framework is practical across a variety of datasets, including those with limited sample sizes, highlighting its flexibility and robustness in adapting to different data sources and clinical conditions.

## CRediT authorship contribution statement

**Tahereh Hassanzadeh:** Writing – original draft, Visualization, Validation, Resources, Methodology, Investigation, Formal analysis, Data curation, Conceptualization. **Sonal Sachdev:** Writing – review & editing, Validation, Data curation. **Wei Wen:** Writing – review & editing, Supervision. **Perminder S. Sachdev:** Writing – review & editing, Validation, Supervision. **Arcot Sowmya:** Writing – review & editing, Validation, Supervision, Project administration, Conceptualization.

## Declaration of competing interest

The authors declare that they have no known competing financial interests or personal relationships that could have appeared to influence the work reported in this paper.

## Data Availability

The data that has been used is confidential.
